# Multiple-Purpose Connectivity Map Analysis Reveals the Benefits of Esculetin to Hyperuricemia and Renal Fibrosis

**DOI:** 10.3390/ijms21207695

**Published:** 2020-10-18

**Authors:** Yiming Wang, Weikaixin Kong, Liang Wang, Tianyu Zhang, Boyue Huang, Jia Meng, Baoxue Yang, Zhengwei Xie, Hong Zhou

**Affiliations:** 1 Department of Pharmacology and Department of the Integration of Chinese and Western Medicine, School of Basic Medical Sciences, Peking University Health Science Center, Beijing 100191, China; wangyiming931211@163.com (Y.W.); wangliang94@bjmu.edu.cn (L.W.); boyueyue960521@126.com (B.H.); mengjia_2011@pku.edu.cn (J.M.); baoxue@bjmu.edu.cn (B.Y.); 2 Peking University International Cancer Institute, Peking University Health Science Center, Beijing 100191, China; 1510307407@pku.edu.cn (W.K.); zty@uw.edu (T.Z.); 3 Department of Molecular and Cellular Pharmacology, School of Pharmaceutical Sciences, Peking University Health Science Center, Beijing 100191, China

**Keywords:** connectivity map, esculetin, hyperuricemia, renal fibrosis, hexokinase 2

## Abstract

Hyperuricemia (HUA) is a risk factor for chronic kidney disease (CKD). Serum uric acid (SUA) levels in CKD stage 3–4 patients closely correlate with hyperuricemic nephropathy (HN) morbidity. New uric acid (UA)-lowering strategies are required to prevent CKD. The multiple-purpose connectivity map (CMAP) was used to discover potential molecules against HUA and renal fibrosis. We used HUA and unilateral ureteral occlusion (UUO) model mice to verify renoprotective effects of molecules and explore related mechanisms. In vitro experiments were performed in HepG2 and NRK-52E cells induced by UA. Esculetin was the top scoring compound and lowered serum uric acid (SUA) levels with dual functions on UA excretion. Esculetin exerted these effects by inhibiting expression and activity of xanthine oxidase (XO) in liver, and modulating UA transporters in kidney. The mechanism by which esculetin suppressed XO was related to inhibiting the nuclear translocation of hexokinase 2 (HK2). Esculetin was anti-fibrotic in HUA and UUO mice through inhibiting TGF-β1-activated profibrotic signals. The renoprotection effects of esculetin in HUA mice were associated with lower SUA, alleviation of oxidative stress, and inhibition of fibrosis. Esculetin is a candidate urate-lowering drug with renoprotective activity and the ability to inhibit XO, promote excretion of UA, protect oxidative stress injury, and reduce renal fibrosis.

## 1. Introduction

Hyperuricemia (HUA) is associated with gout, obesity, hypertension, atherosclerosis, the metabolic syndrome, lipid disorders, cardiovascular disease, and CKD [1,2]. Recent epidemiologic and experimental evidence has identified HUA as a common complication of CKD, and an independent risk factor for CKD development and progression [3]. A reduction in glomerular filtration rate (GFR) contributes to HUA, which is frequently observed in patients with CKD [4]. HUA is an independent risk factor for cardiovascular disease and hypertension, which further contributes to the development and progression of CKD [5]. In addition, because the kidney is responsible for the excretion of UA, the prevalence of HUA in patients with CKD is higher than in the general population [6]. Individuals with HUA (>9 mg/dL) have a three-fold higher risk of CKD development. Furthermore, HUA is associated with a greater incidence of end-stage renal disease (ESRD) [7].

The effective management of HUA is important to delay the development and progression of CKD and reduce cardiovascular complications in hyperuricemic patients. However, difficulties with the efficacy and safety of urate-lowering therapies in patients with CKD, particularly those with intermediate and advanced renal deficiencies, are common [8].

SUA is the terminal oxidation product of purine metabolism in humans, and which is regulated by the kidneys. The kidney is responsible for eliminating 70% of the daily UA production. The renal handling of UA includes glomerular filtration, proximal tubular reabsorption, secretion, and post-secretory reabsorption.

Urate-lowering therapy is central to the management of HUA in gout [9]. Urate-lowering drugs fall into one of three classes: XO inhibitors (allopurinol and febuxostat), uricosurics (URAT1 inhibitors benzbromarone) and recombinant uricases. Amongst them, allopurinol, an XO inhibitor, is the most frequently prescribed agent for gout in the United States [10]. Unfortunately, the majority of patients treated with allopurinol do not achieve target SUA levels, possibly due to an intolerance to allopurinol at doses ≥ 300 mg dose and the need for reduced doses in patients with renal insufficiency [11]. It is therefore recommended to initially evaluate renal function in gout patients with CKD [12]. According to the specific conditions of the patients, treatments with a low impact on renal function should be used to monitor adverse reactions [13,14]. This highlights the need for anti-hyperuricemic agents with greater effectiveness and fewer side effects.

CMAP provides an alternative to drug discovery as it evaluates the function of compounds through transcriptional profiling as opposed to the assessment of protein binding partners. CMAP is advantageous for diseases with large-scale changes in gene expression, multiple phenotypes, and lack of known protein targets. Previously, through the comparison of disease signatures and expression profiles, celastrol has been identified as a potent leptin sensitizer. Multiple gene expression profiles with a common phenotype, obesity, have been combined for CMAP analysis [15]. Herein, we proposed, even far different phenotypes are possible to be countered together by one single compound using different scoring gene sets. In our previous work, thousands of differentially regulated genes were identified in HUA with HN (not shown data), so we reasoned that CMAP could predict small molecules that could correct these gene expression disorders. HUA is typically associated with progressive renal fibrosis. We therefore propose that applications of CMAP using multiple-purpose strategies to target both HUA and renal fibrosis can aid the discovery of novel and more effective compounds targeting a single phenotype.

Based on the multiple-purpose CMAP approach, we discovered a candidate compound, esculetin. Esculetin, is a naturally occurring 6,7-dihydroxy derivative of coumarin that can improve various disease states, including obesity, diabetes, cardiovascular, renal failure, cancer, and neurological disorders. The beneficial effects of esculetin have been ascribed to its antioxidant, anti-proliferative, and cytoprotective effects. In this study, our data show that esculetin treatment could reverse thousands of down- or up-regulated genes by RNA-Seq analysis. 

In addition, we verified the beneficial effects of esculetin on HUA with HN and renal fibrosis, which were consistent with CMAP results, and revealed pathways targeted.

## 2. Results

### 2.1. Esculetin Is the Top Scoring Candidate Compound

**Figure 1:**
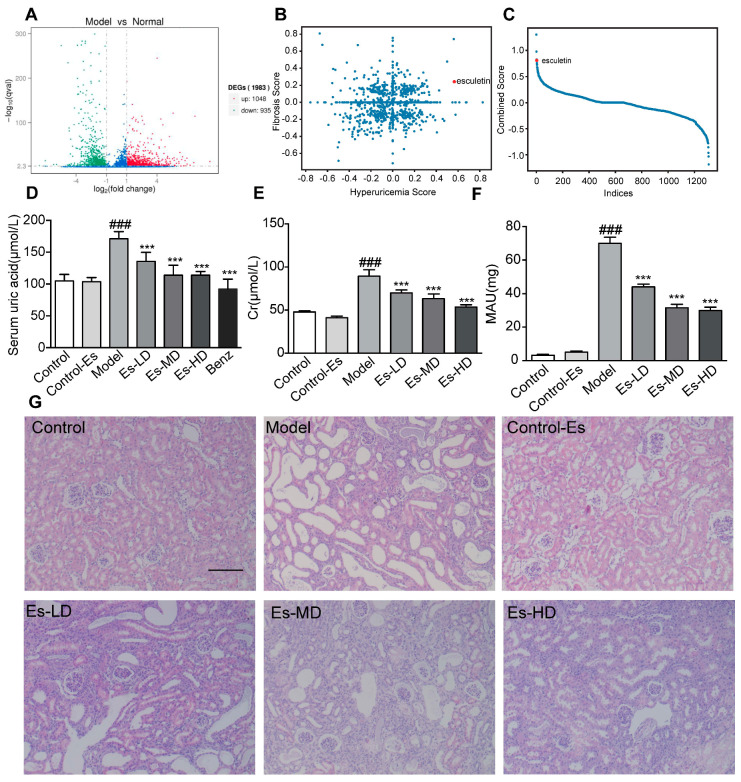
Connectivity map analysis and the effects of Esculetin on HUA with HN. (**A**) Scatter plot of the enrichment scores of HUA and fibrosis. Each dot represents an individual small molecule. Esculetin had a high HUA and positive fibrosis score. (**B**) Sum of the HUA and fibrosis scores. Esculetin had the 4th greatest score amongst the small molecules. *X* axis: individual small molecules. (**C**) The *y*-axis is the sum of Hyperuricemia Score and Fibrosis Score. The *x*-axis is the molecules sorted from high to low according to the Combined Score. (**D**) Serum UA levels in HUA model mice. (**E**) Serum creatinine levels. (**F**) Urine microalbumin. Vertical bars show data with mean ± SD (*n* = 6). ### *p* < 0.001 vs. Control. *** *p* < 0.001 vs. Model. (**G**) Photomicrographs of HE stained kidney tissues of HUA model mice (magnification 200×).

To search for potential candidate compounds that target both HUA with HN and renal fibrosis, we measured the gene expression changes in control and HUA mice using RNA-Seq. Compared to control mice, thousands of differentially expressed genes were identified (Figure 1A). We mapped the mouse genes to human homologues and selected the top 100 differentially expressed genes as the query set. For fibrosis, we compared the expression profiles of a cystic fibrosis cell line and a control cell line [16].

Both queries were submitted to the web tool (https://portals.broadinstitute.org/cmap/). We plotted both the HUA and fibrosis scores (Figure 1B). Scores were multiplied by –1 for clarity. The sum of the scores is shown in Figure 1C. Esculetin ranked 4th and was the first available compound. Experiments were performed to confirm its functions. 

### 2.2. Esculetin Reduces SUA Levels in a Dose-Dependent Manner

As shown in Figure 1D, SUA was elevated in HUA mice. Following esculetin treatment, SUA decreased in a dose-dependent manner. Similar effects were observed for benzbromarone. Serum creatinine (Cr) and proteinuria (MAU) (markers of renal function) were significantly reduced (Figure 1E,F) indicating improved renal function. HE staining showed characteristic histological abnormalities in HUA mice, including epithelial cell disturbances, tubular basement membrane disintegration and tubular dilatation. Esculetin administration preserved the kidney architecture and reduced tubulointerstitial damage (Figure 1G). Esculetin therefore improves renal function and alleviates kidney injury in HUA with HN. As shown in Supplementary Figure S1A, esculetin was unable to improve urea nitrogen (BUN) levels.

### 2.3. Esculetin Inhibits XO Expression and Activity

**Figure 2:**
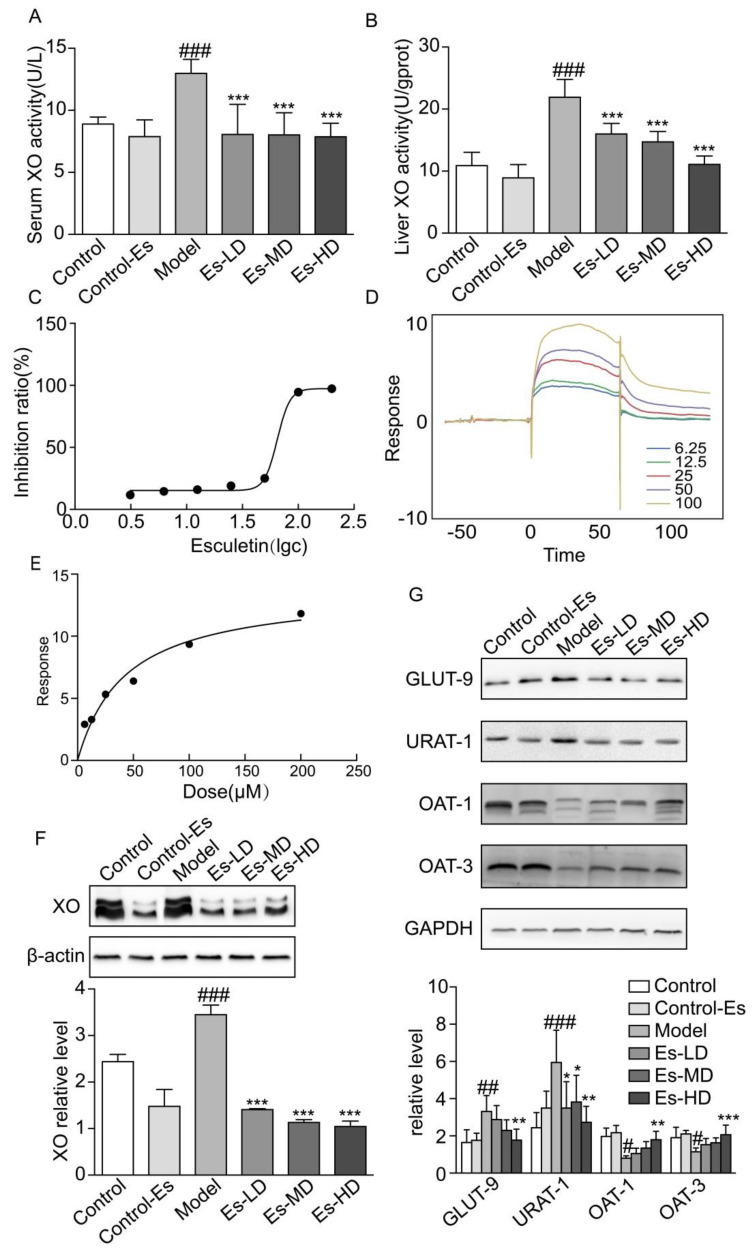
Esculetin reduces SUA levels. (**A**) Serum XO activity of HUA model mice, vertical bars show data with mean ± SD (*n* = 6). (**B**) Liver XO activity. Vertical bars show data with mean ± SD (*n* = 6). ### *p* < 0.001 vs. Control. *** *p* < 0.001 vs. Model. (**C**) Inhibition rates of esculetin on XO in vitro, IC_50_ = 64.93 μM. (**D**) SPR dynamics of esculetin and XO. (**E**) SPR steady-state results of esculetin and XO. (**F**) Expression of XO in the livers of HUA model mice assessed through western blot analysis. (**G**) Uric acid transporter expression in the kidneys of HUA model mice. Vertical bars show data with mean ± SD (*n* = 5). # *p* < 0.05, ## *p* < 0.01 ### *p* < 0.001 vs. Control. * *p* < 0.05, ** *p* < 0.01 and *** *p* < 0.001 vs. Model.

Though target information was not used to predict functional compounds, it was likely that esculetin targets traditional hyperuricemic proteins, including XO or renal UA transporters. In HUA mice, the activity of XO in the liver and serum increased. Treatment with esculetin for 3 weeks effectively inhibited XO activity in both the liver and serum (Figure 2A,B). The IC_50_ of esculetin in vitro was 64.93 μM (Figure 2C). The expression of XO in the liver was also suppressed following esculetin treatment (Figure 2F).

We measured the binding affinity of esculetin to XO using SPR. The response units (RU), as an indicator of affinity, increased in response to increasing esculetin concentrations, suggesting that esculetin binds to XO in a concentration-dependent manner. The equilibrium dissociation constants (KD) of esculetin binding to XO were 5.836 × 10^−5^ (Figure 2D,E). Taken together, these data suggest that esculetin binds directly to XO in vitro.

### 2.4. Esculetin Modulates the Expression of Renal UA Transporters

Renal UA transporters maintain UA balance in the kidney. It has been reported that HUA mice induced by oxonic acid displayed dysregulated urate transporter expression. We found that URAT-1 and GLUT-9 were upregulated, and OAT-1/3 were downregulated in HUA. Following esculetin treatment, these expression profiles were reversed (Figure 2G). Thus, esculetin inhibits UA reabsorption and improves UA excretion.

### 2.5. Esculetin Suppresses Renal Fibrosis in HUA and UUO Mice

**Figure 3:**
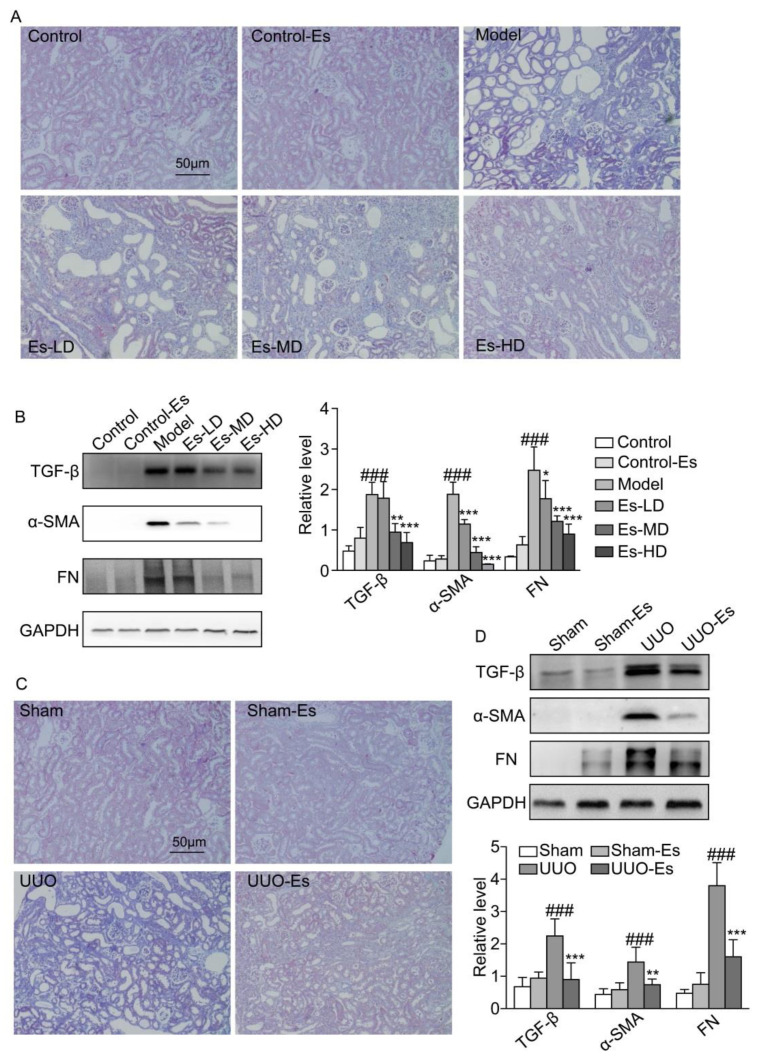
Effects of esculetin on renal fibrosis. (**A**) Photomicrographs of Masson’s trichrome stained kidney tissues in HUA model mice (magnification 200×). (**B**) Western blots of fibrosis-related proteins in HUA model mice. Vertical bars show data with mean ± SD (*n* = 5). ### *p* < 0.001 vs. Control. * *p* < 0.05, ** *p* < 0.01 and *** *p* < 0.001 vs. Model. (**C**) Photomicrographs of Masson’s trichrome stained kidney tissues of UUO model mice (magnification 200×). (**D**) Western blots of fibrosis-related proteins in UUO model mice. Means ± SD (*n* = 5). Vertical bars show data with mean ± SD (*n* = 5). ### *p* < 0.001 vs. Sham. ** *p* < 0.01 and *** *p* < 0.001 vs. UUO.

As predicted by CMAP, esculetin could reduce fibrosis (cs score = −0.243). To confirm this effect, we examined the fibrotic markers TGF-β1, α-SMA and FN. Each marker was upregulated by 3.8-, 10-, and 10-fold respectively in HUA mice, and reduced to normal levels by esculetin (Figure 3B). Masson’s trichrome staining demonstrated collagen accumulation and severe morphologic lesions in HUA kidneys (Figure 3A). Treatment with esculetin reduced these phenotypes. In 7-day UUO models, similar trends were observed for TGF-β1, α-SMA, and FN following Masson’s trichrome staining (Figure 3C,D).

### 2.6. Esculetin Reduces XO-Dependent and NADPH Oxidase-Dependent Renal Oxidative Stress in HUA Mice

**Figure 4:**
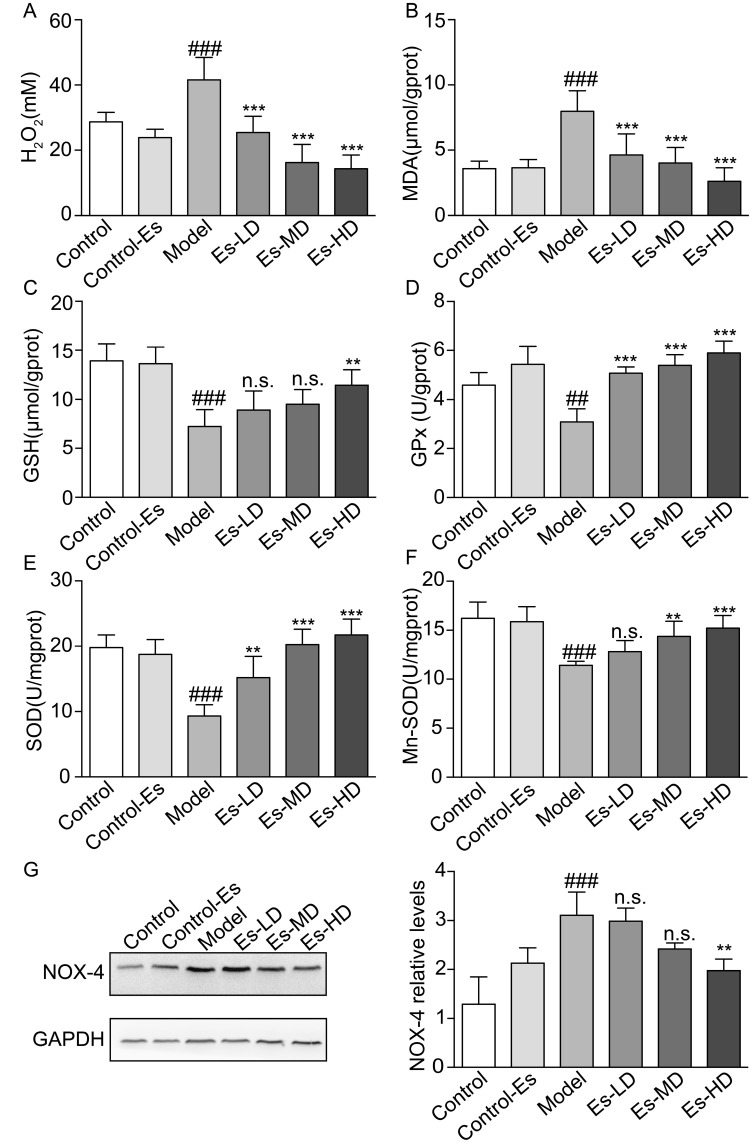
Effects of esculetin on oxidative stress levels in HUA model mice. (**A**) Serum H_2_O_2_ levels. Vertical bars show data with mean ± SD (*n* = 6) (**B**) MDA activity in renal tissue. Vertical bars show data with mean ± SD (*n* = 6) (**C**) GSH levels in renal tissue. Vertical bars show data with mean ± SD (*n* = 6) (**D**) GPx activity in renal tissue. Vertical bars show data with mean ± SD (*n* = 6) (**E**) SOD activity in renal tissue. Vertical bars show data with mean ± SD (*n* = 6) (**F**) Mn-SOD activity in renal tissue. Vertical bars show data with mean ± SD (*n* = 6). *n*.s., not significant. ## *p* < 0.01 and ### *p* < 0.001 vs. Control. ** *p* < 0.01 and *** *p* < 0.001 vs. Model. (**G**). Expression of Nox-4 in the kidney was determined by western blot analysis. Vertical bars show data with mean ± SD (*n* = 5). n.s., not significant. ### *p* < 0.001 vs. Control. ** *p* < 0.01 vs. Model.

XO catalyzes the production of UA, accompanying the production of free radicals. At the same time, the increased blood UA levels activate renal oxidative stress. Esculetin downregulated the serum H_2_O_2_ and renal malondialdehyde (MDA) levels which were elevated in HUA mice (Figure 4A,B). SOD, GSH, and GPx levels were measured to evaluate the effects of esculetin on XO-mediated oxidative stress in the kidney. Compared to the control group, SOD, Mn-SOD, GSH, and GPx levels were suppressed in HUA mice. Three weeks of esculetin treatment effectively reversed these phenotypes in a dose dependent manner (Figure 4C–F). NADPH oxidase 4 (Nox-4) was abundantly expressed in the renal proximal tubule and was upregulated in HUA mice. Esculetin effectively reduced Nox-4 expression to normal levels (Figure 4G). These results suggest that the renal protective effects might be the consequences of reducing XO-dependent and NADPH oxidase-dependent renal oxidative stress.

### 2.7. Esculetin Improves Oxidative Stress Dysfunction and Enhances Nuclear Nrf2 Translocation in NRK-52E Cells

**Figure 5:**
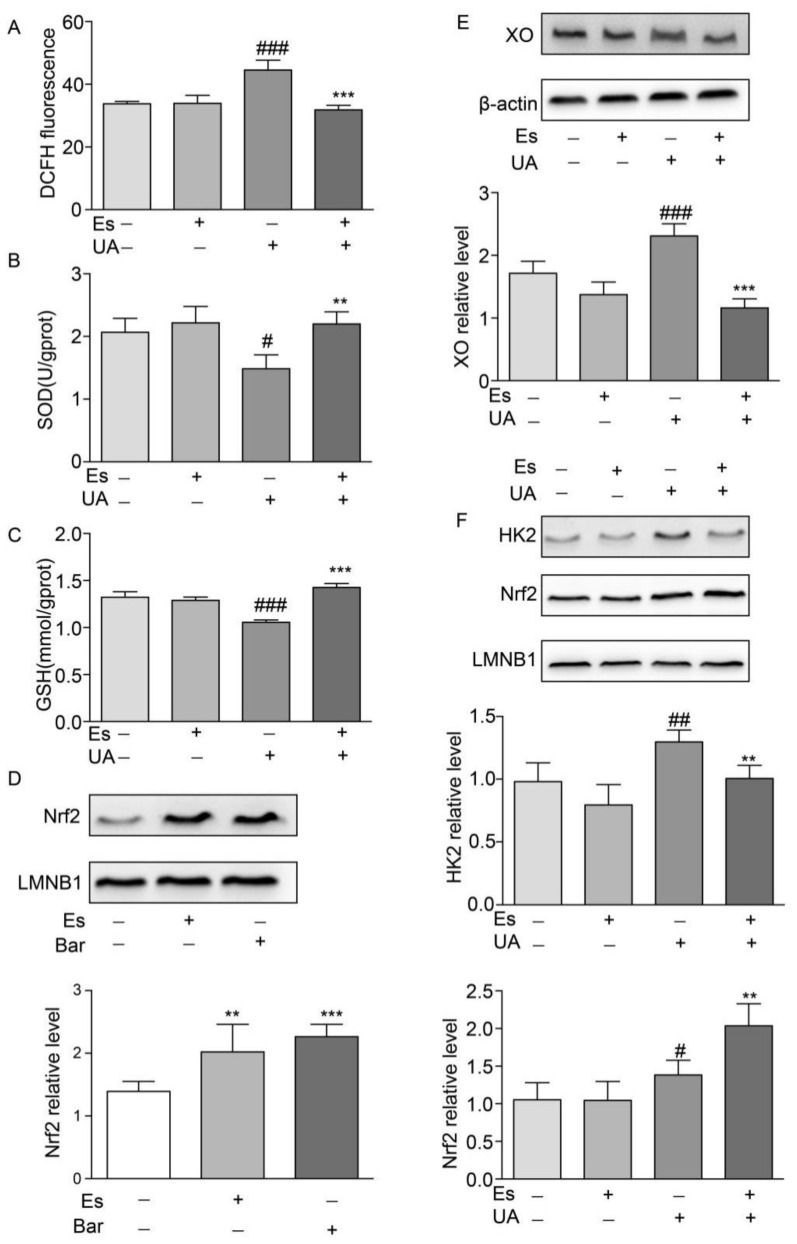
Effects of esculetin on the nuclear translocation of Nrf2 and HK2. (**A**) DCFDA fluorescence. Vertical bars show data with mean ± SD (*n* = 6) (**B**) Effects of esculetin on cellular SOD activity. Vertical bars show data with mean ± SD (*n* = 6) (**C**) Effects of esculetin on cellular GSH levels. Vertical bars show data with mean ± SD (*n* = 6). # *p* < 0.05, ### *p* < 0.001 vs. untreated group. ** *p* < 0.01 and *** *p* < 0.001 compared to UA only. (**D**) Effects of esculetin on nuclear Nrf2 accumulation in NRK-52E cells. Nuclear Nrf2 was determined by Western blot analysis. Vertical bars show data with mean ± SD (*n* = 5). ** *p* < 0.01 and *** *p* < 0.001 vs. untreated group. (**E**). Western blots of XO in HepG2 cells following UA irritation ± esculetin treatment. (**F**) Western blots of Nrf2 and HK2 in nuclear fractions from HepG2 cells. Vertical bars show data with mean ± SD (*n* = 5). # *p* < 0.05, ## *p* < 0.01, ### *p* < 0.001 vs. untreated group. * *p* < 0.05, ** *p* < 0.01 and *** *p* <0.001 compared with UA treated group.

To confirm the protective mechanisms of esculetin against oxidative stress, we irritated cells using high concentrations of UA (400 μM) for 12 h and treated cells with esculetin (9 μM) for 14 h (including a 2 h pre-treatment). ROS levels were measured through DCFH fluorescence. We found that UA exposure moderately increased intracellular ROS production which decreased in response to esculetin treatment (Figure 5A). SOD and GSH were measured as indicators of the antioxidant defense systems in NRK-52E cells following oxidative stress. Notable decreases in intracellular SOD and GSH following UA induction were observed which recovered following esculetin treatment (Figure 5B,C). This suggests that esculetin enhances the antioxidant capability of NRK-52E cells. 

To investigate the effects of esculetin on Nrf2 translocation in NRK-52E cells, cells were treated with esculetin (9 μM) or bardoxolone methyl (Bar) as a positive control (10 nM) for 12 h. Figure 5D shows that treatment with esculetin significantly increases Nrf2 levels in the nuclear fraction.

### 2.8. Esculetin Down-Regulates HK2 in UA-Induced HEPG2 Cells

UA administration increased the expression of XO which decreased in response to esculetin treatment (Figure 5E). It has been reported that Nrf2 transcriptionally activates XO in glioma cells, which is dependent on Nrf2 and HK2 trafficking to the nucleus [17]. Esculetin treatment abolished the translocation of HK2 to the nucleus whilst the nuclear import of Nrf2 was upregulated in UA-treated HepG2 cells (Figure 5F). These results suggest that esculetin decreases the expression of XO through inhibiting the nuclear translocation of HK2 in UA-induced HEPG2.

### 2.9. Esculetin Reverses the Transcriptome Alterations 

**Figure 6:**
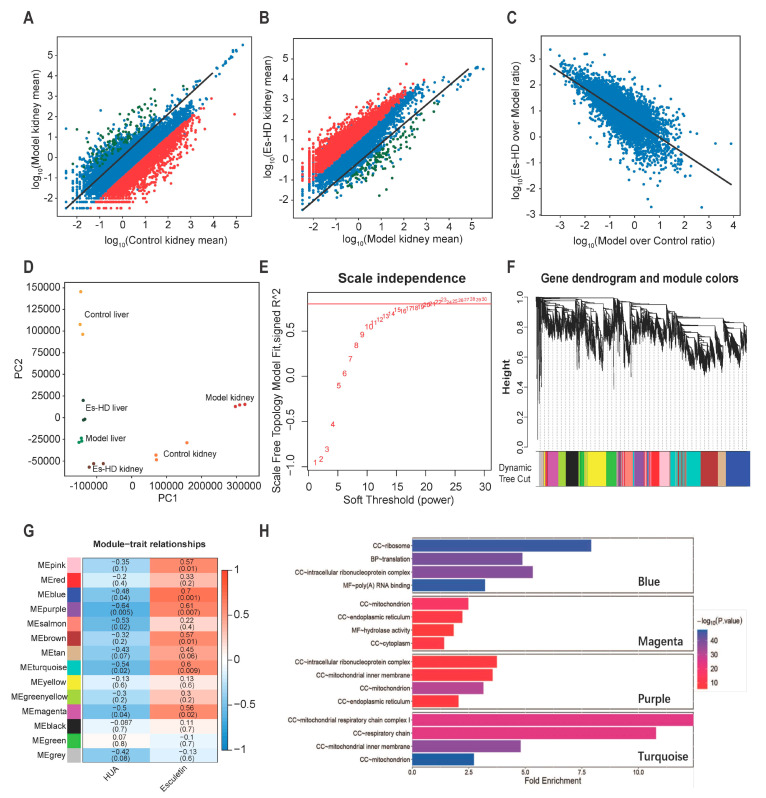
RNA-seq analysis reveals transcriptional reversion by esculetin in the kidney. (**A**) Scatter plots of the gene expression in Model vs. Control groups. Black line: *y* = *x* line. (**B**). Scatter plots of gene expression in Es-HD versus Model groups. Red (green) dots show genes that were down- (up-) regulated by 100 fold (**A**) which were revered after esculetin treatment (**B**) Black line: *y* = *x* line. (**C**) Scatter plots of gene expression in Model vs. Controls and Es-HD vs. Model ratios in the log_10_ scale. Changes in the ratios indicated a perfect restoration. (**D**) PCA analysis of 18 samples using absolute gene expression data in the kidney and liver. The distance between different dots indicates their similarity. (**E**) *x*-axis: different power value; *y*-axis: r-square value after linear regression of -log10 (k) and log10 (*p* (k)). K: connectivity of the gene nodes; *p* (k): possibility of such a node. Horizontal line: 0.8. (**F**) The result of gene clustering. Different colors show different gene modules. (**G**) Gene modules and phenotypes quantified using the Boolean variable (1 represents occurrence, 0 represents no occurrence) were used to calculate the correlation coefficient, which is demonstrated as a heat map. *p*-values are shown in brackets. (**H**) The results of GO analysis in blue, magenta, purple, and turquoise modules. Among them, “BP” stands for biological process, “CC” stands for cell component, and “MF” stands for molecular function.

To evaluate the effects of esculetin on the transcriptome changes observed, we performed RNA-seq of the liver and kidney in Control, Model and Es-HD mice. In HUA model mice, we confirmed our previous findings (not shown data) in which ≥1000 genes were up- or down-regulated in the kidney. Up to 3000 genes were down-regulated 10-fold. In response to esculetin, the repression of the genes was reversed in the kidney (>95% and 90% for 10- and 500-fold respectively) (Figure 6A–C). In the liver, we observed further changes in gene expression (Supplementary Figure S2A) and a similar reversion was observed in response to esculetin (Supplementary Figure S2B,C). These reversions were evident via principle component analysis, in which esculetin-treated kidneys/liver displayed transcriptomes that were comparable to control models (Figure 6D). It is noteworthy that esculetin also enhanced the expression of specific genes in the kidney (Figure 6B,C). 

To analyze the gene modules that regulated these changes, weighted gene correlation network analysis (WGCNA) [18] was performed by “WGCNA” package in R (4.0.2) [19]. Those genes whose average expression level in all samples were less than 0.5, or whose median absolute deviation were less than 1.5 were eliminated, and a total of 8833 genes were finally included in WGCNA. To meet the conditions of the scale-free network, we selected a soft threshold = 22, the R square reached 0.8 (Figure 6E). In the clusters (marked by different colors in Figure 6F), we identified the highest levels of repression or reversion. As shown in Figure 6G, blue, purple, turquoise, and magenta groups were repressed to the highest levels (bluer, *p* < 0.05) and reverted following treatment by Esculetin significantly (*p* < 0.05). The representation of each gene in the module and the relationship between the gene and phenotype were presented as scatter plots (Supplementary Figure 2D–G). It was generally believed that genes with genetic significance (GS) greater than 0.5 and molecular membership (MM) greater than 0.8 had close connections and key functions with other genes in the module. There were many genes in the blue, purple, turquoise and magenta modules that satisfy GS > 0.5 and MM > 0.8, which indicated that the genes in these four modules were closely related and may have similar functions. After that, we conducted gene ontology analysis in these four modules. It can be seen from the results (Figure 6H) that the genes in magenta and purple modules were related to the function of mitochondria and the genes in turquoise ere closely related to the respiratory chain in mitochondria. HUA usually caused mitochondrial oxidative damage and functional changes, which showed that the use of esculetin can reverse the expression of related genes.

## 3. Discussion

In the clinic, the combination of XO inhibitors and uricosurics is a promising approach in patients failing to reach target SUA levels following XO monotherapy [20]. In this study, we highlight the potential of esculetin as a urate-lowering drug, through its ability to inhibit XO and promote UA excretion. Furthermore, esculetin is more suitable for gout and hyperuricemic patients with renal deficiency/CKD due to its ability to reduce oxidative stress and renal fibrogenesis in HUA mice.

The homeostasis of SUA is mainly governed by hepatic production and renal excretion. XO is the rate-limiting enzyme during purine metabolism [21]. We therefore investigated the potential of esculetin as an effective XO inhibitor. In in vivo experiments, esculetin could inhibit the expression and activity of liver XO in HUA mice. In in vitro experiments, SPR suggested that esculetin could bind XO directly in a concentration-dependent manner (Figure 2D,E). The IC_50_ of esculetin on XO in vitro was 64.93 µM.

Inefficient renal excretion of UA is the most common cause of HUA in gout patients [22]. Uricosuric drugs tend to control the renal excretion of UA through inhibiting renal tubular reabsorption, thus reducing SUA levels [23]. The increased excretion of UA down-regulates URAT1, and GLUT9 and upregulates OAT1 and OAT3. These results suggest that esculetin is a potent dual-function urate-lowering agent.

UA-lowering therapy plays an important role in delaying the progression of CKD. Tubulointerstitial fibrosis is a common feature of various kidney diseases which are associated with CKD progression to ESRD. A higher UA level is associated with a significant decline in eGFR and a higher risk of kidney failure [24,25]. In vivo, UA displays both an anti- and pro-oxidant role, which is dependent on the biological conditions [26]. Emerging evidence suggests that chronic HUA contributes to hypertension, cardiovascular complications, and kidney disease [27,28]. The pathophysiologic mechanisms of HN include oxidative stress, endothelial dysfunction, vasoconstriction, and stimulation of the renin angiotensin aldosterone system (RAAS) [29]. It is well known that oxidative stress strongly influences CKD [30]. In recent years, an emerging body of evidence shows that UA-induced oxidative stress is central to tubular injury and tubulointerstitial fibrosis in HUA mice [31,32,33]. Combined with the CMAP data, we therefore examined the effects of esculetin on oxidative stress and renal fibrosis in HN.

Superoxide anions are generated in HUA model mice by XO or NADPH oxidase. During the oxidative hydroxylation of xanthine to UA, XO generates ~25% O^2−^ and ~75% H_2_O_2_. NOX-4 is the predominant form of NADPH oxidase in the kidney and is abundantly expressed as a source of ROS. It has been demonstrated that UA increases intracellular ROS production by activating NOX-4 in human tubular cells [34]. Thus, in this study, we investigated H_2_O_2_ production in the serum and ROS production in the renal tissue. In HUA model mice, we found that elevated SUA and ROS in the blood and kidney were related to renal dysfunction, including increased serum creatine, BUN, and proteinuria in SUA conditions. However, ROS overproduction was suppressed is response to esculetin (Figure 4). These results suggest that esculetin treatment for up to three weeks can significantly ameliorate renal function through the suppression of SUA and oxidative stress. 

In addition, the renal histopathological lesions in HUA mice significantly improved (Figure 1F and Figure 2A). Moreover, Masson’s staining results showed that renal fibrosis occurred in the model mice and esculetin administration significantly prevented the development and progression of renal fibrosis. Amongst the numerous fibrogenic factors, transforming growth factor-β 1(TGF-β1) is essential to the onset and progression of renal fibrosis. Targeting TGF-β1 activated signaling pathways is a potentially effective therapy for renal fibrosis [35,36]. In this study, we demonstrated that esculetin treatment significantly suppressed TGF-β1-dependent profibrotic signaling in HUA mice. To further confirm our CMAP findings, we then created a second renal fibrosis model (UUO) [37]. Masson’s staining and TGF-β1- signaling was comparable to that observed in HUA mice. Our data therefore suggests that CMAP is a useful tool to identify novel candidate compounds. 

Esculetin is a coumarin compound. To date, ≥1300 coumarin compounds have been isolated from natural organisms which are widely distributed in plants. Some animals and microorganisms produce coumarin compounds [38]. Coumarins possess antioxidative activity [39,40] and esculetin is a well-established antioxidant [41,42,43]. Previous studies demonstrated that esculetin could bind to cytoplasmic Kelch-like ECH associated protein 1 (Keap1) to promote the nuclear translocation of Nrf2 in pancreatic cancer cells [44]. Elevated Nrf2 levels in the nucleus induces antiproliferative and antiapoptotic responses possibly through the attenuation of NF-*k*B. Nrf2 is a basic leucine zipper and redox-sensitive transcription factor that is a master regulator of antioxidant transcription, enhancing the cytoprotection against oxidative stress [45]. Under stress conditions, Nrf2 dissociates from Keap1 and enters the nucleus to attach to the promoter region of ARE (antioxidant response element) [46]. To examine the antioxidative mechanisms of esculetin in HN, we determined the effects of esculetin on Nrf2 and oxidative stress in the renal proximal tubule cell line NRK-52E. We found that esculetin promotes the translocation of Nrf2 to the nucleus and improves SOD and GSH activity. Combined with the in vivo data, we therefore suggest that the renal protective effects of esculetin are related to Nrf2-mediated antioxidant responses in the kidneys of HUA mice. 

XO is also a target of Nrf2. When the effects of esculetin on Nrf2 nuclear translocation in HepG2 cells was investigated, a similar phenotype to NRK-52E cells was observed. This was surprising since we were unable to explain the molecular mechanisms by which XO was inhibited. It has previously been shown that HK2 acts as a transcriptional coactivator of Nrf2 to regulate XO expression. Decreased nuclear translocation in response to HK2 may therefore inhibit XO expression [17]. We therefore hypothesize that the molecular mechanisms by which esculetin suppresses XO is related to its ability to inhibit the nuclear translocation of HK2 (Figure 5F).

In this study, we used benzbromarone as a positive control agent. Benzbromarone is a uricosuric agent and non-competitive inhibitor of XO that is used to treat gout when the first line treatment (allopurinol) produces adverse effects [47]. Benzbromarone is associated with fulminant hepatitis, liver failure, and rare cases of serious liver toxicity [48,49,50]. Previous studies have showed that the hepatotoxicity of esculetin is negligible [51,52,53,54]. In this study, hepatic damage was assessed by through AST and ALT activity. Our results indicate that esculetin causes no hepatotoxicity in HUA mice.

## 4. Materials and Methods 

### 4.1. Connectivity Map Analysis

RNA-Seq was performed in HUA model and control groups. Genes were ranked by fold changes. Mouse genes were mapped to human genes through homologue mapping. The top 100 altered genes were used as queries in the CMAP website (https://portals.broadinstitute.org/cmap/). 

### 4.2. WGCNA

Gene co-expression network analysis exponentially ranks the correlation coefficients of genes to obtain an adjacency matrix. This allows the genetic network to follow that of a scale-free network. A topological matrix that incorporates the surrounding genetic information was calculated using the distance d.

(1) $$S_{ij}=\mathrm{cor}\left(i,j\right)$$

(2) $$\alpha_{ij}={\left|S_{ij}\right|}^{\beta }$$

(3) $$\omega_{ij}=\frac{l_{ij}+\alpha_{ij}}{\min\left\{k_{i},k_{j}\right\}+1-\alpha_{ij}}$$

(4) $$d_{ij}=1-\omega_{ij}$$

The $l_{ij}=\underset{u}{\sum }\alpha_{iu}\alpha_{uj}$, and $k_{i}=\underset{u}{\sum }\alpha_{iu}$ represent the node connectivity. We built a clustering tree and used the dynamic pruning method to determine the gene module (maxBlocksize = 5000, deepSplit = 2, minModuleSize = 30). We obtained the Pearson correlation coefficients between different gene modules and phenotypes. Gene enrichment analysis was performed on gene modules with higher correlation coefficients to obtain key biological processes. We calculated the Pearson correlation coefficients between the genes and modules, and between the gene and the phenotype. 

### 4.3. Chemical Agents

Esculetin was purchased from Cayman Chemicals (Ann Arbor, MI, USA). Hypoxanthine and potassium oxonate were purchased from Sigma-Aldrich (St. Louis, MO, USA). Bardoxolone methyl was purchased from Selleck Chemicals (Shanghai, China).

### 4.4. Animal Experiments and Ethics

Healthy 7~8-week-old ICR male mice in the HUA mouse model and healthy 7~8-week-old male C57BL/6J mice weighing approximately 18 g to 20 g in unilateral ureteral occlusion (UUO) model were obtained from the Department of Laboratory Animal Science, Peking University Health Science Center (Beijing, China). Mice were housed in open top conventional cages with poplar bedding material in a pathogen free environment. A maximum of five mice were housed in a single cage. All mice were exposed to a 12-h light/dark cycle under defined environmental conditions at 25 ± 2 °C with a relative humidity of 50%. All mice were given free access to food and water. All animal care and experimental procedures complied with the Animals (Scientific procedures) Act 1986 and all procedures involving animals were confirmed to follow the Regulations for the Administration of Affairs Concerning Experimental Animals published by the State Science and Technology Commission of China. The study was approved by the Biomedical Ethics Committee of Peking University. Animal studies were reported in compliance with ARRIVE guidelines (Kilkenny, Browne, Cuthill, Emerson, Altman & Group, 2010; McGrath & Lilley, 2015).

### 4.5. HUA Mouse Models

ICR male mice (7~8 weeks old) were acclimatized to the Animal Center of Peking University Health Science Center for at least 1 week prior to the experiments. The HUA mouse model was established by peritoneal injection of a mixture of hypoxanthine (300 mg·kg^−1^) and potassium oxonate (300 mg·kg^−1^) daily for 2 weeks. Control mice were treated with saline. Animals were euthanized and the kidneys and livers collected for protein analysis and histological examinations. Blood was obtained for the measurement of serum uric acid, BUN, creatinine, and other biochemistry indices. 

To explore the preventive effects of esculetin on HUA mice, mice were randomly divided into control (Control), control-operated esculetin-treated (Control-Es), HUA mouse models (Model), high dose esculetin (Es-HD), middle dose esculetin (Es-MD), and low dose esculetin groups (Es-LD). Mice in the Control and Model groups were orally administered 200 μL 0.5% CMC-Na once per day. Mice in the Sham-Es and the Es-H received esculetin at a dose of 300 mg·kg^−1^ body weight, whilst Es-M and Es-L received 100 and 33.33 mg·kg^−1^ body weight, respectively, through oral administration once per day. To analyze its efficacy, esculetin was administered for 3 weeks, including a 1-week pretreatment. Benzbromarone was administered as a positive control (Benz, 50 mg·kg^−1^ orally once per day).

### 4.6. UUO Model

C57BL/6J male mice (7~8 weeks old) were acclimatized to the Animal Center of Peking University Health Science Center for at least 1 week prior to experiments. For the UUO model, mice underwent ligation of the left ureter and were sacrificed on the 7th day. Sham operated mice underwent laparotomy and exposure of the left ureter. Animals were euthanized, and the kidneys and livers were collected for protein analysis and histological examination. Blood was taken for the measurement of serum uric acid, BUN, creatinine, and other biochemical indices.

To explore the preventive effects of esculetin on UUO mice, mice were randomly assigned to the sham-operated group (Sham), sham-operated esculetin-treated group (Sham-Es), UUO group (UUO) and high dose esculetin group (Es-UUO). Mice in the Sham and Model groups were orally administered 200 μL 0.5% CMC-Na once per day. Mice in the Sham-Es and Es-H received esculetin at a dose of 300 mg·kg^−1^ body weight by oral administration once per day. To analyze the efficacy of esculetin, it was administered for the full 2 weeks, including a 1-week pretreatment. 

### 4.7. Assessment of UA, Renal Function, Liver Function and Other Biochemical Indices

Serum UA, creatinine, BUN, serum AST, serum ALT, kidney GSH and hydrogen peroxide were determined using commercially available kits (NJJC Bio, Nanjing, China) according to the manufacturer’s instructions. Kidney GPx, SOD and Mn-SOD were measured using kits obtained from Beyotime Biotechnology (Shanghai, China). Microalbuminuria was assessed by ELISA (Wuhan Xinqidi Biological Technology Co., Ltd. Wuhan, China).

### 4.8. SPR Biosensor Analysis

The binding affinity of baicalein to XO in vitro was assayed using the SPR-based Biacore 3000 instrument (Biacore AB, Uppsala, Sweden). XO (molecular mass, 160 kDa) was purchased from Sigma-Aldrich. XO protein was immobilized onto a CM5 sensor chip according to standard procedures. Data were collected at a constant HBS-EP flow rate of 30 μL/min at 25 °C. Esculetin was diluted into the running buffer to create a series of concentrations from 100 μM to 6.25 μM. Samples were injected into the channels at a flow rate of 30 μL/min and the binding responses were continuously recorded in RU. The association (Kon) and dissociation (Koff) rate constants and equilibrium dissociation constants (KD) were calculated.

### 4.9. XO Activity and IC_50_

Esculetin/DMSO (1000 mM) were diluted to 100 μM in 0.01 M PBS. Different concentrations of esculetin (20 μL volume) were mixed with 50 μL 60U/L XO solution at 25 °C for 20 min. Using XO activity kits to determine enzyme activity in the mixture, the inhibition rates in the presence of esculetin were determined. XO activity were determined using commercial kits (NJJC Bio, Nanjing, China) according to the manufacturer’s protocol.

### 4.10. Cell Culture

HepG2 and NRK-52E cells were purchased from the Cell Resource Center of Shanghai Institutes for Biological Sciences, Chinese Academy of Sciences. Cells were maintained in Dulbecco’s modified Eagle’s medium (DMEM) supplemented with 10% (*v*/*v*) heat-inactivated fetal bovine serum, 1% non-essential amino acids, 1% sodium pyruvate, 100 U/mL penicillin, and 100 μg/mL streptomycin in a humidified incubator with 5% CO_2_ at 37 °C. Cells were grown to 50% confluency prior to treatment. Esculetin was dissolved in DMSO and added to the culture medium. Cell and nuclear proteins were extracted using commercially available kits (Bestbio Science, Beijing, China).

For ROS assessment, cells were pre-treated with 9 µM esculetin for 2 h in the presence and absence of 15 mg/dL UA for 24 h. Cells were stained with 10 µM DCFDA for 20 min at room temperature in the dark. The fluorescent intensity of DCF was measured at 515 nm by flow cytometry (10,000 events per sample). SOD activity and GSH levels were determined using commercial kits (Beyotime Biotechnology and NJJC Bio) according to the manufacturer’s instructions. 

### 4.11. Western Blots Analysis

Cells were lysed in RIPA lysis buffer, and nuclear proteins were extracted using commercial kit (BestBio Science, Beijing, China). Equal amounts of protein were subject to 10% SDS-PAGE and transferred to polyvinylidene difluoride (PVDF) membranes (Millipore Corp., Danvers, MA, USA). Membranes were blocked and probed with primary antibodies against GAPDH, XO, LMNB1, URAT1 (Proteintech Group, Chicago, IL, USA), fibronectin (Abcam, Cambridge, UK), TGF-β (Cell Signaling Technology, Beverly, MA, USA), MMP-7, MMP-9, Nox-4, α-SMA, Nrf2, HK2, OAT-3 (ABclonal Technology, Wuhan, China), OAT-1 (Abbkine Scientific Co., Ltd. Wuhan, China), and GLUT-9 (Novus Biologicals) overnight at 4 °C. Membranes were labeled with goat anti- rabbit IgG or goat anti-mouse IgG (Santa Cruz, Dallas, TX, USA) HRP-conjugated secondary antibodies for 45 min at room temperature. Membranes were washed and exposed using an ECL plus kit (Amersham Biosciences, Buckinghamshire, UK). Images were scanned with an Epson scanning system, and data were analyzed using Quantity-one software. In order to ensure the validity of the experimental results, all Western blot results have been added to Supplementary Figure 3.

### 4.12. Statistical Analyses

All data are presented as mean ± SD. Data were analyzed using a two-tailed Student’s *t*-test (for two-grouped comparisons) or ANOVA (for multiple-group comparisons). Significance was assigned at *p* < 0.05. * *p* < 0.05, ** *p* < 0.01, *** *p* < 0.001. n.s., not significant. In all figures, *n* referred to the sample size which was selected based on previous studies. Unless otherwise indicated, the results were based on a minimum of five independent experiments to ensure reproducibility. Statistical analyses were performed using GraphPad Prism software.

## 5. Conclusions

In conclusion, our data show that esculetin effectively lowers the SUA and ameliorates renal tubulointerstitial fibrosis in HUA mice. The urate lowering effects are mediated through reduced UA production by XO inhibition, and increased urate excretion by mediating transporter expression. The renoprotection of esculetin is correlative with lowering UA, inhibiting oxidative stress and TGF-β-activated fibrosis.

## Supplementary Materials

Supplementary Materials can be found at https://www.mdpi.com/1422-0067/21/20/7695/s1. Figure S1. (A) Serum urea nitrogen levels in HUA model mice. (B) Serum AST. (C) Serum ALT. Mean ± SD (*n* = 6). n.s., not significant. ## *p* < 0.01 and ### *p* < 0.001 vs. Control. Figure S2. RNA-seq analysis reveals transcriptional reversion by esculetin in liver. The function of the gene was analyzed. (A) Scatter plots of gene expression in mice of the Model group vs. the Control group. Black line: *y* = *x* line. (B) Scatter plot of gene expression in the mouse model group vs. Es-HD. Red (green) dots shows genes that were down- (up-) regulated by 100 fold (A) which were reversed following esculetin treatment (B). Black line: *y* = *x* line. (C) Scatter plots of the Model over Control ratios vs. Es-HD over the model group in the log10 scale. The changes in the ratios indicated a perfect restoration. (D) Blue module gene correlation scatter plots. *X*-axis represents the molecule membership (MM), i.e., the Pearson’s correlation coefficient of the gene and module. *Y*-axis represents the genetic significance (GS) for the phenotype, i.e., the Pearson’s correlation coefficient of the gene and phenotype (phenotype is represented by a Boolean variable). (E) Purple module gene correlation scatter plots. (F) Turquoise module gene correlation scatter plots. (G) Magenta module gene correlation scatter plots. Figure S3 shows all results of Western blot.

## Abbreviations

HUA	Hyperuricemia
CKD	chronic kidney disease
SUA	serum uric acid
HN	hyperuricemic nephropathy
UA	uric acid
CMAP	connectivity map
UUO	unilateral ureteral occlusion
XO	xanthine oxidase
HK2	hexokinase-2
GFR	glomerular filtration rate
ESRD	end-stage renal disease
RNA-Seq	RNA sequencing
Cr	creatinine
MAU	proteinuria
BUN	urea nitrogen
RU	response units
MDA	malondialdehyde
SOD	superoxide dismutase
GSH	reduced glutathione
GPx	glutathione peroxidase
Nox-4	NADPH oxidase 4
ROS	reactive oxygen species
Bar	Bardoxolone methyl
Keap1	Kelch-like ECH associated protein 1
Nrf2	Nuclear factor-erythroid-2 related factor
ARE	antioxidant response element
AST	aspartate aminotransferase
ALT	alanine transaminase
