# Supplementary material for: Multiple-Purpose Connectivity Map Analysis Reveals the Benefits of Esculetin to Hyperuricemia and Renal Fibrosis
Source: Int J Mol Sci. 2020 Oct 18;21(20):7695. doi: 10.3390/ijms21207695 (PMC7589473; doi:10.3390/ijms21207695)
Supplement: Supplementary file 1 [file ijms-21-07695-s001.pdf]

International Journal of
Molecular Sciences

MDPI

Supplementary Materials

Multiple-purpose Connectivity Map Analysis Reveals the Benefits of Esculetin to Hyperuricemia and Renal Fibrosis

Yiming Wang [1,†], Weikaixin Kong [2,3,†], Liang Wang [1], Tianyu Zhang [2], Boyue Huang [1], Jia Meng [1], Baoxue Yang [1], Zhengwei Xie [2,*] and Hong Zhou [1,*]

[1] Department of Pharmacology and Department of the Integration of Chinese and Western Medicine, School of Basic Medical Sciences, Peking University Health Science Center, Beijing 100191, China; wangyiming931211@163.com (Y.W.); wangliang94@bjmu.edu.cn (L.W.); boyueyue960521@126.com (B.H.); mengjia_2011@pku.edu.cn (J.M.); baoxue@bjmu.edu.cn (B.Y.)

[2] Peking University International Cancer Institute, Peking University Health Science Center, Beijing 100191, China; zty@uw.edu

[3] Department of Molecular and Cellular Pharmacology, School of Pharmaceutical Sciences, Peking University Health Science Center, Beijing 100191, China; 1510307407@pku.edu.cn

* Correspondence: xiezhengwei@hsc.pku.edu.cn (Z.X.) rainbow_zhou@126.com (H.Z.); Tel.: +86-10-82802798 (Z.X.); +86-10-82802798 (H.Z.)

† These authors equally contributed to this work.

Received: 23 August 2020; Accepted: 3 October 2020; Published: 18 October 2020

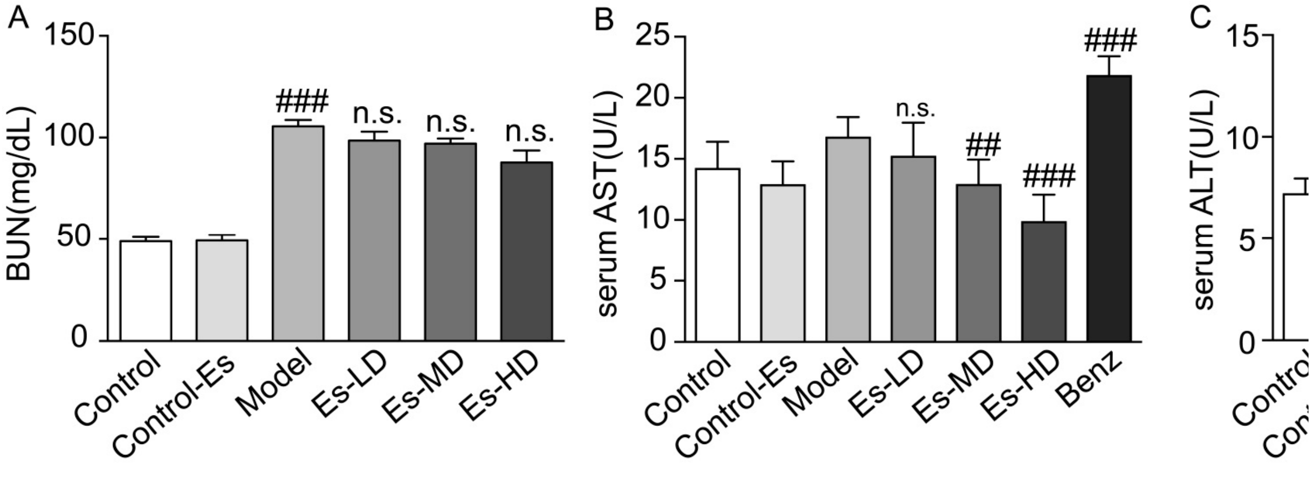

Figure S1. (**A**) Serum urea nitrogen levels in HUA model mice. (**B**) Serum AST. (**C**) Serum ALT. Mean ± SD (n=6). n.s., not signi

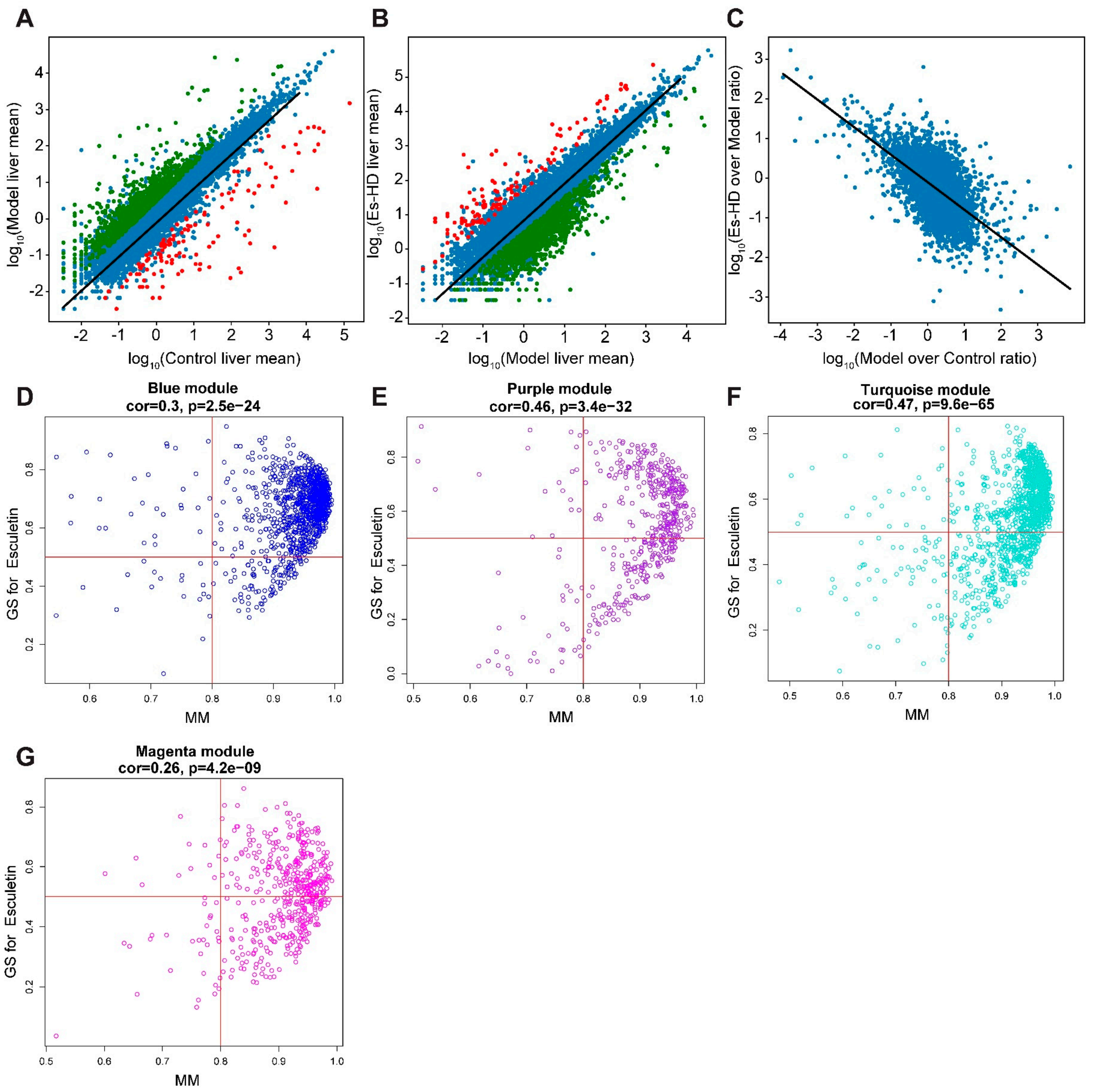

Figure S2. RNA-seq analysis reveals transcriptional reversion by esculetin in liver. The function of the gene was analyzed. (**A**) Scatter plots of gene expression in mice of the Model group vs the Control group. Black line: *y*=*x* line. (**B**) Scatter plot of gene expression in the mouse model group vs Es-HD. Red (green) dots shows genes that were down- (up-) regulated by 100 fold (A) which were reversed following esculetin treatment (B). Black line: *y*=*x* line. (**C**) Scatter plots of the Model over Control ratios vs Es-HD over the model group in the log10 scale. The changes in the ratios indicated a perfect restoration. (**D**) Blue module gene correlation scatter plots. *X*-axis represents the molecule membership (MM), i.e. the Pearson's correlation coefficient of the gene and module. *Y*-axis represents the genetic significance (GS) for the phenotype, i.e. the Pearson's correlation coefficient of the gene and phenotype (phenotype is represented by a Boolean variable). (**E**) Purple module gene correlation scatter plots. (**F**) Turquoise module gene correlation scatter plots. (**G**) Magenta module gene correlation scatter plots.

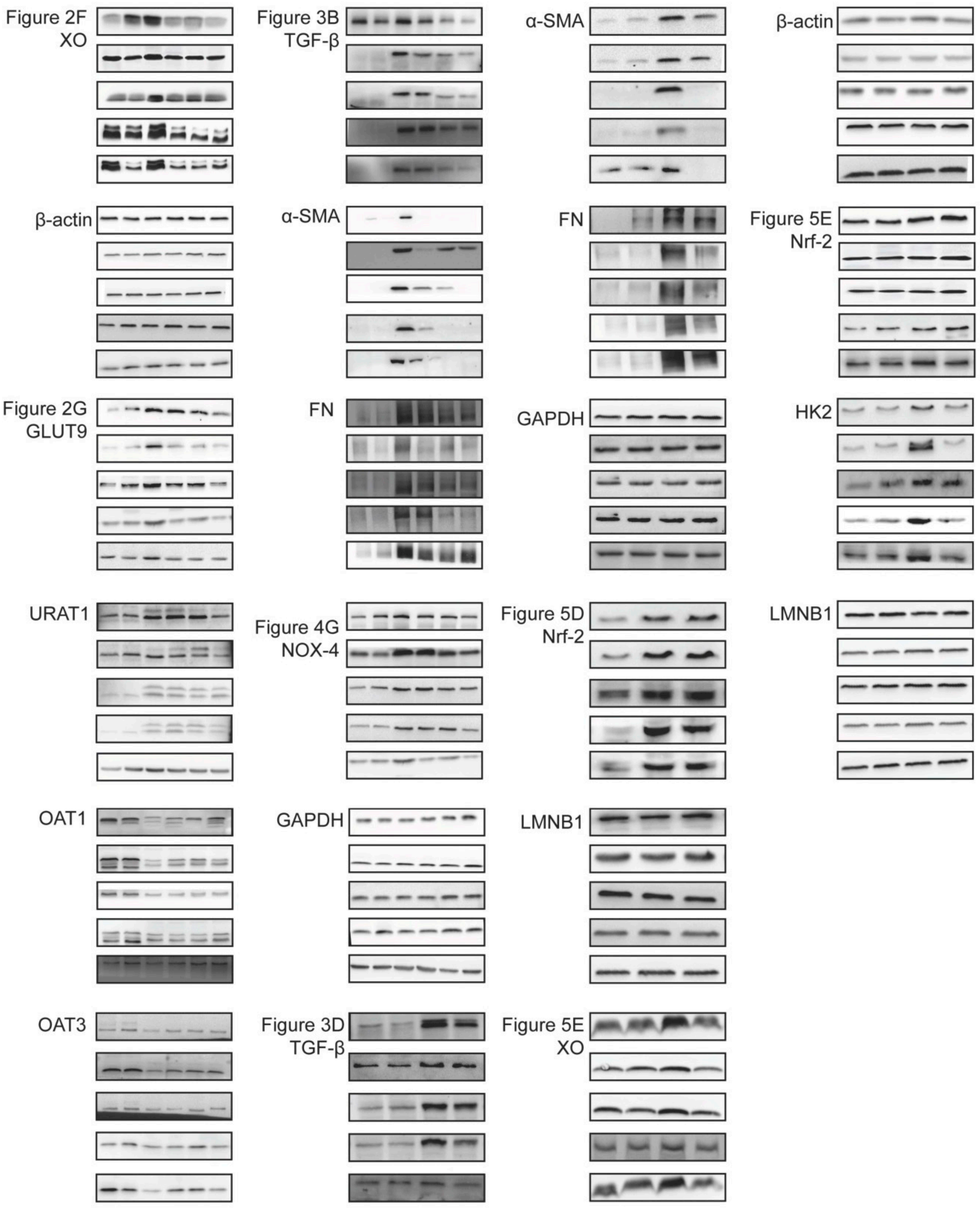

Figure S3. All results of Western blot.
